# Limited Agreement between Classifications of Diabetes and Prediabetes Resulting from the OGTT, Hemoglobin A1c, and Fasting Glucose Tests in 7412 U.S. Adults

**DOI:** 10.3390/jcm9072207

**Published:** 2020-07-13

**Authors:** Larry A. Tucker

**Affiliations:** College of Life Sciences, Brigham Young University, Provo, UT 84602, USA; tucker@byu.edu

**Keywords:** hyperglycemia, glycated hemoglobin, NHANES, blood sugar, sensitivity, specificity

## Abstract

This investigation was designed to determine the degree of concordance resulting from tests of fasting plasma glucose (FPG) and hemoglobin A1c (A1c) compared to the oral glucose tolerance test (OGTT) for detecting prediabetes and diabetes in undiagnosed adults. Another objective was to measure concordance within subsamples of women and men, and within three age groups. Lastly, the value of combining the FPG and A1c for detecting diabetes was compared to the OGTT. A total of 7412 randomly selected adults from the National Health and Nutrition Examination Survey (NHANES) were included. With outcomes classified as normal, prediabetes, or diabetes, according to standard guidelines, overall test agreements were low. With an OGTT diagnosis of diabetes, concordance was only 34% for the A1c assessment and 44% for the FPG assay. Delimited to older adults, agreement between the OGTT and A1c was only 25%, and between the OGTT and FPG, concordance was only 33.5%. Given the large percentage of discordant results associated with the FPG and A1c, clinicians should be cautious about employing these tests as lone assessments. Using both the FPG and A1c helped with accurately diagnosing diabetes and normal glycemia, but not prediabetes. The OGTT is a good choice to reduce misdiagnosis.

## 1. Introduction

Nearly a half-billion individuals have diabetes, almost 10% of the world’s adults [1]. By 2045, the prevalence is expected to reach 700 million. In 2019, more than four million deaths resulted from diabetes and its complications [1]. Clearly, diabetes is one of the most devastating global diseases.

Many individuals with diabetes have not been diagnosed. According to the International Diabetes Federation, one-half of those with diabetes do not know they have the disease [1]. As a result, treatment is delayed, and health risks are increased. Millions of other adults have impaired glucose tolerance or prediabetes. Collectively, diabetes, undiagnosed diabetes, and prediabetes have created one of the most significant worldwide public health challenges. 

The human and economic costs associated with diabetes are staggering. Consequently, significant efforts are expended each year with the goal of improving the management of diabetes. However, before diabetes can be successfully treated, it must be accurately diagnosed. 

The 2019 “Standards of Medical Care in Diabetes” by the American Diabetes Association (ADA) [2] lists three methods for diagnosing diabetes: (1) fasting plasma glucose (FPG), (2) the oral glucose tolerance test (OGTT), or (3) hemoglobin A1c (A1c). The ADA document also indicates that these same three tests may be employed to detect prediabetes.

Well-accepted criteria have been established for differentiating among those with diabetes, prediabetes, or normal glucose metabolism. The 2019 ADA report states that the FPG, OGTT, and A1c are each appropriate for diagnosing diabetes, generally. However, research shows that agreement between the FPG and the OGTT is less than impressive, and classifications based on the glucose-based protocols and A1c are often not in alignment. Research shows that the OGTT tends to diagnose more individuals with diabetes or prediabetes than the FPG or A1c tests [3].

Because accurate diagnosis is critical for prompt and appropriate treatment of diabetes and prediabetes, a variety of investigations have been conducted to determine the relationship between outcomes produced by the OGTT, FPG, and the A1c assays [4,5,6,7,8]. Results have been mixed. Moreover, few of the studies have had good external validity, and the findings of some are now outdated. 

Specifically, Saukkonen et al. focused on “intermediate hyperglycemia,” defined by an A1c of 5.7–6.4%, along with impaired FPG and OGTT [4]. The scientists studied 486 adults from Finland. They concluded that agreement among the tests was “uncommon.” Similarly, Guo et al. conducted a study with emphasis on the value of the HbA1c assessment for the diagnosis of diabetes and prediabetes in U.S. adults [5]. The FPG and OGTT were employed for comparison purposes. They found low sensitivity and high specificity rates leading to high false-negative results (65–75%). Cowie et al. used NHANES data (2003–2006) and focused on the prevalence of diagnosed and undiagnosed diabetes in the United States [6]. Prevalence rates associated with the A1c, FPG, and OGTT assays were also compared. The authors concluded that using A1c criteria resulted in “substantially lower prevalences of undiagnosed and total diabetes” compared to the glucose-based tests [6].

Combining data from numerous countries, the Non-communicable Disease Risk Factor Collaboration studied diabetes rates in over 300,000 individuals [7]. To be included in the study, participants had to have results from at least two of the three diagnostic tests: A1c, FPG, or OGTT. For the diagnosis of diabetes, using either the OGTT or the FPG test as the referent, the pooled results found a sensitivity of 30.5% for the A1c assay and specificity was nearly 100%. The authors indicated that the three tests resulted in significantly different estimates of diabetes throughout the world [7]. Lastly, Briker et al. studied diabetes rates in 441 African-born blacks [8]. The investigation focused on the OGTT and A1c tests. The overall prevalence of diabetes was 7%. The A1c test alone detected diabetes in 32% of the participants, whereas the OGTT alone identified 68%. The title of the authors’ paper and their conclusion were similar, “A1c underperforms as a diagnostic test in Africans…” [8].

The primary aim of the current study was to evaluate the extent of agreement resulting from diabetes tests of FPG and A1c compared to the OGTT in 7412 previously undiagnosed, randomly selected adults, representing non-institutionalized individuals living in the United States. A secondary purpose was to identify the degree of concordance among the three diagnostic tests for prediabetes. Another objective was to determine the extent of concordance within subsamples of women and men, and within young, middle-age, and older adults. Lastly, the value of employing both the FPG and A1c together for identifying diabetes and prediabetes was compared to the OGTT.

## 2. Materials & Methods

### 2.1. Sample

Data for the present investigation were obtained from the ongoing National Health and Nutrition Examination Survey (NHANES) [9]. NHANES is administered by the U.S. Centers for Disease Control and Prevention (CDC) under the direction of the National Center for Health Statistics. The survey is conducted by the CDC to evaluate the health and lifestyle of individuals living within the country. A complex, multi-level sampling strategy is employed so findings can be generalized nationwide. 

NHANES gathers data using two-year cycles. The present study employed data from the 2009–2010, 2011–2012, 2013–2014, and 2015–2016 cycles. All the NHANES data sets are available online for free [9]. Data derived from the OGTT, A1c, and FPG assays for the NHANES 2017–2018 cycle were not yet available and therefore could not be incorporated into the present study. 

Participants were 20–80 years of age. To be included in the study, subjects had to have complete data for age, sex, the OGTT, A1c, and FPG. 

Adults who reported they were on medication to control their blood sugar were excluded from the sample (*n* = 1170). Women who were pregnant were also excluded (*n* = 14). A total of 164 adults refused to drink or did not consume all the OGTT glucose solution, and another 18 were excluded because they reported they did not fast for at least 9 h, the minimum NHANES used for the FPG test. There were 66 excluded because they came late or left early, 42 who had unsuccessful venipunctures, 16 who were faint during the blood draw, 2 who refused venipuncture, and 551 who were excluded by NHANES for “other” reasons.

Prior to the fasting blood glucose test, subjects were given a fasting questionnaire to assess potential issues associated with fasting [10]. The fasting questionnaire asked about the consumption of coffee or tea, alcohol, gum, mints, lozenges, cough drops, antacids, laxatives, antidiarrheals, and dietary supplements during the fasting period. As part of the current study, participants who reported consuming a prohibited item while fasting were compared to those who reported no intake of any food or beverage, except water, to determine if fasting blood glucose levels were affected by intake of the prohibited item. There were no differences in fasting blood glucose levels associated with any of the items, except alcohol. Therefore, subjects who reported consuming alcohol while “fasting” were excluded from the study (*n* = 44).

There were 7412 participants, 3809 women and 3603 men, who fasted, completed the 3 required blood tests, and fulfilled the other study requirements. Written consent was obtained from each subject. Data collection was carried out following the rules of the Declaration of Helsinki. The Ethics Review Board of the National Center for Health Statistics approved data collection and posting the data online for public use (Protocols #2005–06 and #2011–17) [11]. 

### 2.2. Methods

The key variables of the present study were outcomes of the oral glucose tolerance test (OGTT), the hemoglobin test (A1c), and the fasting plasma glucose test (FPG). Data on age and sex were used to subdivide the sample. 

#### 2.2.1. Race

The following categories were used by NHANES to define race: Mexican American, Non-Hispanic black, Non-Hispanic white, Other Hispanic, and Other race or Multi-racial.

#### 2.2.2. Height and Weight

A Mettler-Toledo digital scale was employed to measure body weight. Each day, the scale was calibrated using precise calibration weights. During the assessment, subjects wore only their underwear, a disposable paper gown, and foam slippers [12]. A stadiometer was used to measure standing height. Height was assessed with both feet flat on the floor and toes angled outwards. Heels, buttocks, shoulder blades, and the back of the head were required to be against the wall [12]. Body weight and height were measured in the NHANES Mobile Examination Center (MEC).

#### 2.2.3. Oral Glucose Tolerance Test and the Fasting Glucose Test

The NHANES quality control and quality assurance protocols met the 1988 Clinical Laboratory Improvement Act mandates. Plasma specimens were processed, stored and shipped to Fairview Medical Center Laboratory at the University of Minnesota, Minneapolis, Minnesota, U.S.A., for analysis. Glucose levels were measured by the hexokinase method [13]. It is an endpoint enzymatic method with a sample blank correction. According to NHANES, “using this enzymatic method, glucose is converted to glucose-6-phosphate (G-6-P) by hexokinase in the presence of ATP, a phosphate donor. Glucose-6-phosphate dehydrogenase then converts the G-6-P to gluconate-6-P in the presence of NADP+. As the NADP+ is reduced to NADPH during this reaction, the resulting increase in absorbance at 340 nm (secondary wavelength = 700 nm) is measured” [13]. This is an endpoint reaction that is specific for glucose. Detailed specimen collection and processing instructions are discussed in the NHANES Laboratory/Medical Technologists Procedures Manual, available online [14].

A fasting glucose blood test was performed on all participants who were examined in the morning session, after fasting at least 9 h. After the initial blood draw, which was used to assess fasting glucose levels, participants were asked to drink a 75-g dose of glucose, Trutol^TM^. A second venipuncture was performed 2 h (±15 min) after drinking the Trutol^TM^ solution.

There were no changes to equipment, lab methods, or the lab site associated with the OGTT and the FPG test until the 2015–2016 NHANES cycle [13]. During prior years, the Cobas C501 analyzer (Roche Diagnostics, Basel, Switzerland) was employed to measure glucose levels. NHANES then changed to the Cobas C311 analyzer. To equalize the results, NHANES compared a random sample of 165 individuals using both machines. They determined that the C311 device produced glucose levels that were 2% higher than the values from the Cobas C501 analyzer, although the two machines had equal variations. A weighted Deming regression was employed to adjust the 2015–2016 plasma glucose values so they matched the results derived from the C501 analyzer. The correlation between the bridging measurements was 0.999 (*p* < 0.0001) [13].

#### 2.2.4. Hemoglobin A1c

Similar to the OGTT and FPG test, blood samples were processed, stored and shipped to the Fairview Medical Center Laboratory at the University of Minnesota, Minneapolis, Minnesota, USA for analysis of A1c. There were no changes to equipment, lab methods, or the lab site associated with the A1c test during the eight years included in the present investigation. The Tosoh Automated Glycohemoglobin Analyzer HLC-723G8 (South San Francisco, CA, USA) was used to measure A1c [15]. According to NHANES, “in this assay, the stable (SA1c) and labile (LA1c) A1c forms can be individually resolved on the chromatogram without manual pretreatment, allowing accurate measurement of the stable form of HbA1c. The analyzer dilutes the whole blood specimen with a hemolysis solution, and then injects a small volume of the treated specimen onto the HPLC analytical column. Separation is achieved by utilizing differences in ionic interactions between the cation exchange group on the column resin surface and the hemoglobin components. The hemoglobin fractions (A1c, A1b, F, LA1c, SA1c, A0 and H-Var) are subsequently removed from the column material by step-wise elution using elution buffers each with a different salt concentration. The separated hemoglobin components pass through the photometer flow cell where the analyzer measures changes in absorbance at 415 nm. The analyzer integrates and reduces the raw data, and then calculates the relative percentages of each hemoglobin fraction” [15]. Comprehensive blood sample collection and treatment instructions are discussed in the NHANES Laboratory Procedures Manual, which is available online [14].

### 2.3. Diabetes Classifications

In their 2019 “Standards of Medical Care” recommendations, the American Diabetes Association (ADA) states that diabetes may be diagnosed using the FPG, OGTT, or the A1c assay [2]. The ADA document also indicates that the same assessments can be used to diagnose prediabetes [2]. 

Well-accepted criteria have been established for the diagnosis of diabetes: (1) FPG ≥ 126 mg/dL (7.0 mmol/L). (2) For the OGTT, two-hours after consuming a 75-g glucose load dissolved in water, a FPG level ≥ 200 mg/dL (11.1 mmol/L). (3) A1C ≥ 6.5% (48 mmol/mol). Using the same tests, prediabetes is defined as (1) FPG 100–125 mg/dL (5.6–6.9 mmol/L), (2) OGTT at 2 h: 140–199 mg/dL (7.8–11.0 mmol/L), (3) A1C: 5.7–6.4% (39–47 mmol/mol) [2].

### 2.4. Statistical Analysis

NHANES assigns each participant a person-level sample weight, which allows findings to be generalized to all civilian, non-institutionalized adults living in the United States. The individual sample weights provided by NHANES represent the unequal probability of selection, nonresponse corrections, and adjustments for independent population controls [16]. 

For the current study, sample weights were based on 8 years of the OGTT records, as recommended by NHANES. SAS SurveyMeans was used to generate weighted means and SAS SurveyFreq was utilized to provide weighted frequencies, including agreement between diabetes classifications for the OGTT, A1c, and FPG. SAS SurveyFreq, with the Jackknife variance method, was used to calculate weighted Kappa. Sensitivity and specificity were used to describe the diagnostic accuracy of the FPG and A1c tests compared to the OGTT. Sensitivity and specificity were calculated using weighted prevalence rather than sample size within each cell of the 2 × 2 matrices. The statistical analyses were calculated using SAS Version 9.4 (SAS Institute, Inc., Cary, NC, USA). 

## 3. Results

Mean (±SE) age of the sample was 46.2 ± 0.3 years and the range was 20–80 years. Means (±SE) for the OGTT, A1c, and FPG were 115.0 ± 0.6 mg/dL (6.4 ± 0.03 mmol/L), 5.5 ± 0.01 %, and 100.1 ± 0.3 mg/dL (5.6 ± 0.02 mmol/L), respectively. The Pearson correlation between the OGTT and the FPG test was 0.67 (*p* < 0.0001), whereas the OGTT and A1c were correlated 0.61 (*p* < 0.0001). The correlation between the A1c and FPG was 0.71 (*p* < 0.0001). 

The sample was comprised of 3809 women and 3603 men, a total of 7412 adults. The racial composition of the sample, as defined by NHANES, was 66.8% non-Hispanic white; 11.0% non-Hispanic black; 8.4% Mexican American; 7.7% Other or multi-race, and 6.0% Other Hispanic. Approximately 25% of the participants were from each two-year sampling cycle: 2009–2010, 2011–2012, 2013–2014, and 2015–2016.

Adults who were taking medication to control their hyperglycemia were not included in the comparisons of the 3 blood tests. However, in a separate analysis, it was determined that 1.6% of the young adults in the sample were taking diabetes medication, whereas 9.9% of middle-aged adults and 20.5% of older adults reported taking medication to control their diabetes. Table 1 displays a range of percentiles, including the median (50th percentile), for the key continuous variables for women and men considered separately and for all subjects combined. 

### 3.1. Comparing the OGTT and A1c

For each analysis comparing the OGTT and the A1c, the OGTT was used as the referent. As shown in Table 2, with all subjects combined together (*n* = 7412) and with the OGTT and the A1c outcomes classified as normal, prediabetes, or diabetic according to standard guidelines, the weighted Kappa (±SE) was 0.355 ± 0.015. A total of 34.1% of participants classified as diabetic according to the OGTT were also classified as diabetic according to their A1c findings. Of adults determined to be prediabetic by the OGTT, 46.1% were also defined as prediabetic using the A1c test. Adults defined by their OGTT results as normal were mostly found to be classified as normal by their A1c results, with 80.7% concordance. With the OGTT and A1c outcomes summarized using a simple 2 × 2 matrix (non-diabetic or diabetic for the OGTT and the A1c), rather than the 3 × 3 matrix displayed in Table 2, sensitivity was 34.1% and specificity was 99.6%. 

As shown in Table 2, agreement between the OGTT and the A1c classifications of normal and prediabetes were almost identical for women and men. However, concordance was lower for women defined as diabetic than men classified as diabetic, with agreement levels of 29.5% for women and 39.8% for men. Weighted Kappa (±SE) for the 3 × 3 matrix shown in Table 2 comparing the OGTT and the A1c results for women was 0.346 ± 0.016. For men, weighted Kappa (±SE) was 0.364 ± 0.024. Using a 2 × 2 matrix, with classifications of non-diabetic or diabetic, sensitivity was 29.5% for women only and specificity was 99.5%. With the sample limited to men, sensitivity was 39.8% and specificity was 99.6%, after accounting for individual sample weights.

Table 3 shows the level of agreement between the OGTT classifications and the A1c results by age group, 20–39, 40–59, and 60–80 years. Among young adults (*n* = 2669), those found to be diabetic using the OGTT were classified as diabetic using the A1c test less than half the time, with 46.4% agreement. Agreement between classifications of prediabetes for both tests was much lower, however, with 26.1% concordance. Individuals classified as normal by the OGTT were also likely to be labeled normal by the A1c assessment, with 90.3% concordance. Weighted Kappa (±SE) for young adults for the OGTT and the A1c was 0.277 ± 0.030. Based on a 2 × 2 matrix, with subjects classified as non-diabetic or diabetic, sensitivity was 46.4% and specificity was 99.9%, after taking into account individual sample weights.

Among middle-age adults (*n* = 2617), as shown in Table 3, individuals diagnosed as diabetic using the OGTT were also identified as diabetic by the A1c assessment 42.1% of the time. Those labeled prediabetic by the OGTT were also classified as prediabetic using the A1c test 45.9% of the time. There was 77.8% overlap for those identified as normal using the OGTT and normal according to the A1c assessment. The weighted Kappa (±SE) results for middle-age adults comparing agreement between the OGTT and the A1c was 0.330 ± 0.025. Displayed as a basic 2 × 2 matrix, with categories of non-diabetic or diabetic, sensitivity was 42.1% and specificity was 99.5%, based on calculations performed using NHANES sample weights.

As shown in Table 3, older adults (*n* = 2126) who were labeled diabetic by the OGTT were also labeled diabetic by the A1c assessment 25.2% of the time. Agreement for classifications of prediabetes was 55.8% and overlap for classifications of normal was 64.0%. For older adults, the weighted Kappa (±SE) results comparing the OGTT and the A1c in the 3 × 3 matrix shown in Table 3 was 0.301 ± 0.024. Based on a 2 × 2 matrix, with participants identified as either non-diabetic or diabetic, sensitivity was 25.2% in older adults and specificity was 99.1%, after applying individual sample weights.

### 3.2. Comparing the OGTT and the Fasting Plasma Glucose Test (FPG)

Focusing on the total sample (*n* = 7412), as displayed in Table 4, with the OGTT and the fasting plasma glucose (FPG) outcomes categorized as normal, prediabetes, or diabetes, the weighted Kappa (±SE) was 0.310 ± 0.011. A total of 44.3% of the adults identified as diabetic according to their OGTT were also diagnosed as diabetic based on their FPG results. Adults labeled prediabetic by the OGTT were typically defined as prediabetic using the FPG, with 63.4% agreement. Adults defined by their OGTT results as normal were usually classified as normal also by their FPG results, with 64.8% concordance. With the OGTT and the FPG classifications summarized using a simple 2 × 2 matrix (i.e., non-diabetic or diabetic), sensitivity was 44.3% and specificity was 98.7%, based on results derived using individual sample weights and all participants.

As shown in Table 4, concordance between the OGTT and the FPG classifications of normal, prediabetes, and diabetes were different for men and women. Agreement based on diagnoses of diabetes using the OGTT, followed by FPG results also concluding diabetes, was higher in men than women, with 57.9% concordance compared to 33.7% agreement. For classifications of prediabetes, agreement was higher in men than women, with 59.2% concordance in women and 68.0% in men. Concordance was higher for women defined as normal compared to men classified as normal, with OGTT and FPG agreement levels of 73.4% for women and 55.3% for men. 

Weighted Kappa (±SE) for the 3 × 3 matrix shown in Table 4 comparing the OGTT classifications and the FPG results for women was 0.351 ± 0.016. For men, weighted Kappa (±SE) was 0.274 ± 0.019. Using a straightforward 2 × 2 matrix, with classifications of non-diabetic or diabetic, sensitivity was 33.7% for women and specificity was 99.2%, calculated using NHANES sample weights. With the sample delimited to men, sensitivity was 57.9% and specificity was 98.1%, after accounting for individual sample weights.

Table 5 shows the degree of concordance between the OGTT classifications and the FPG outcomes by age group. Among young adults (*n* = 2669), 20–39 years old, those diagnosed as diabetic using the OGTT were labeled diabetic using the FPG test about half the time, with 54.0% agreement. Concordance between classifications of prediabetes for both tests was slightly lower, with 52.4% concordance. Young adults labeled normal by the OGTT were frequently labeled normal by the FPG assessment, with 73.8% agreement. The weighted Kappa statistic (±SE) for individuals representing young adults for the OGTT and the FPG was 0.206 ± 0.025. Using a basic 2 × 2 matrix, with subjects classified as non-diabetic or diabetic for the OGTT and FPG test, sensitivity was 54.0% and specificity was 99.5%, after accounting for individual sample weights.

Among middle-age adults (*n* = 2617), 40–59 years old, as shown in Table 5, those diagnosed as diabetic using the OGTT were also identified as diabetic by the FPG test 56.1% of the time. Individuals labeled prediabetic by the OGTT were also classified as prediabetic using the FPG assessment with 62.6% concordance. There was 60.4% overlap for those identified as normal using the OGTT and normal according to the FPG test. The weighted Kappa (±SE) results for middle-age adults based on the 3 × 3 matrix shown in Table 5 was 0.294 ± 0.019. Displayed as a 2 × 2 matrix, with categories of non-diabetic or diabetic, sensitivity was 56.1% and specificity was 98.3%, based on calculations performed using NHANES sample weights.

As shown in Table 5, older adults (*n* = 2126), 60–80 years old, who were labeled diabetic by the OGTT were also categorized as diabetic by the FPG test 33.5% of the time. Agreement for classifications of prediabetes was 69.5% and concordance for classifications of normal was 51.9%. For older adults, the weighted Kappa (±SE) results comparing the OGTT and the FPG assessment in the 3 × 3 matrix shown in Table 5 was 0.310 ± 0.022. Based on a simple 2 × 2 matrix, with participants identified as either non-diabetic or diabetic, sensitivity was 33.5% in older adults and specificity was 97.6%, after applying NHANES sample weights.

**Figure 1:**
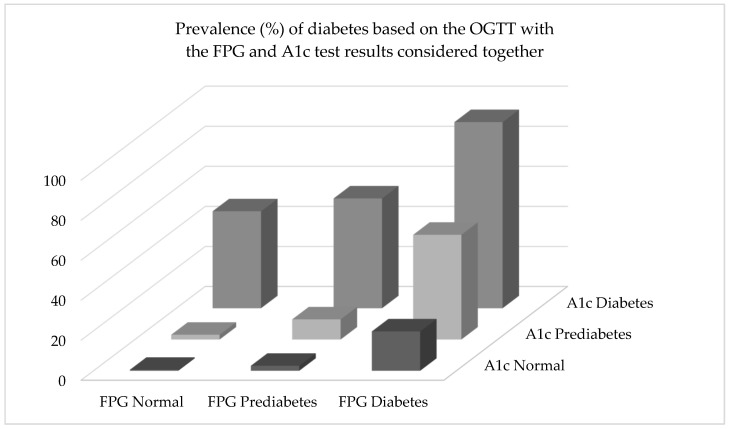
For the 2-h OGTT, “Normal” was defined as a plasma glucose level of <140 mg/dL (<7.8 mmol/L), “Prediabetes” was 140–199 mg/dL (7.8–11.0 mmol/L), and “Diabetes” was a glucose level ≥200 mg/dL (11.1 mmol/L). For the FPG (fasting plasma glucose) assessment, “Normal” was <100 mg/dL (<7.0 mmol/L), “Prediabetes” was 100–125 mg/dL (5.6–6.9 mmol/L), and “Diabetes” was defined as a glucose level ≥126 mg/dL (≥7.0 mmol/L). For the A1c test, “Normal” was <5.7% (<39 mmol/mol), “Prediabetes” was 5.7–6.4% (39–47 mmol/mol), and “Diabetes” was an A1c ≥ 6.5% (≥48 mmol/mol). An example of interpretation of the results would be as follows: Prevalence of diabetes, based on the OGTT, was 54.9% among adults classified as having prediabetes by the FPG test and diabetes by the A1c test.

Figure 1 displays a 3-dimensional model of the relationship between the combined classifications of the FPG and A1c tests and the prevalence of diabetes defined by the OGTT. The results were based on the hypothetical arrangement that patients were screened using both the FPG and A1c, not just one or the other. Findings showed that about 20% of adults were diagnosed with diabetes (OGTT) when the FPG assay indicated a classification of diabetes and the A1c concluded a normal test. With a FPG label of normal and an A1c classification of diabetes, 48.5% were determined to be diabetic. With the FPG test indicating diabetes and the A1c assay pointing to prediabetes, 52.4% were found to be diabetic. When both the FPG and A1c tests identified individuals as diabetic, the OGTT also classified adults as diabetic 93% of the time. Although not shown in Figure 1, when both the FPG and A1c assessments classified individuals as normal, agreement with the OGTT was the same, 93%.

Using the FPG and A1c together to identify prediabetes resulted in low levels of agreement with the OGTT (not shown). Specifically, when the FPG test showed normal results and the A1c test indicated prediabetes, the OGTT indicated prediabetes 15.4% of the time. With the classifications flipped for the FPG and A1c tests, 16.8% of adults were identified as prediabetic. When both the FPG and A1c assays concluded prediabetes, 32.4% of adults were classified as prediabetic by the OGTT. 

## 4. Discussion

The primary purpose of the present investigation was to determine the extent of agreement between classifications of normal, prediabetes, and diabetes, based on the OGTT, A1c, and FPG blood tests using standard cut-points. Another aim was to assess concordance between diabetes classifications within women and men separately, and within young, middle-age, and older adults separately. Another objective was to determine the effects of employing both the FPG and A1c together for detecting diabetes and prediabetes when compared to the OGTT. Because the NHANES sample was randomly selected using a sophisticated, multi-level design, and each statistical analysis was performed accounting for strata, clusters, and individual sample weights, the results can be generalized to all the civilian, non-institutionalized U.S. adult population.

Compared to the FPG and A1c tests, numerous investigations show that the OGTT is the best single measure for predicting subsequent diabetes, disease, and/or mortality [17,18,19,20,21]. Although more time-consuming and difficult to administer for the diagnosis of diabetes, the OGTT is considered by many to be the gold standard [22,23,24,25,26,27,28]. Consequently, in the present study, the OGTT was used as the referent in each comparison.

### 4.1. Comparing the OGTT and A1c

Findings showed that classification agreement between the OGTT and the A1c test was generally poor, especially for the identification of diabetes. When the entire sample of 7412 adults was analyzed and the OGTT resulted in a classification of diabetes, the A1c test agreed only 34.1% of the time. In other words, approximately two-thirds of the time when the OGTT indicated diabetes, the A1c did not. When the sample was delimited to women, the A1c assessment agreed with the OGTT only 29.5% of the time. Clearly, the two blood tests are not in harmony when the outcome is a diagnosis of diabetes. Similarly, there was low concordance when the OGTT resulted in a classification of prediabetes. The A1c test agreed less than half the time (46.1%).

When the sample was confined to specific age groups, young adults, middle-age adults, and older adults, agreement between the OGTT and A1c was not good within any of the age groups. Alignment was especially poor within the older adult group, 60–80 years old, when the OGTT resulted in a classification of diabetes. The A1c assessment agreed only 25.2% of the time. In short, 75% of the time when the OGTT indicated diabetes, the A1c test classified the individual as normal or prediabetic, not diabetic.

A close look at the research used to validate the A1c as a measure of chronic plasma glucose may help explain the discrepancies between the OGTT and A1c. The first major study used data from the Diabetes Control and Complications Trial (DCCT) [29]. Rohlfing et al. employed linear regression weighted by the number of observations per subject to correlate mean plasma glucose and A1c findings. The resulting regression equation, derived from monitoring 1439 individuals, showed that A1c accounted for 67% of the variance in mean chronic plasma glucose levels. Moreover, according to the regression equation derived from the trial, the 95% prediction error for an average subject was ±69 mg/dL (±3.81 mmol/L) when participants had A1c levels between 6% and 9% (42–75 mmol/mol) [29]. Most would consider this margin of error too large to be useful. 

Because of the high level of prediction error associated with the DCCT equation, in 2008, Nathan et al. used data from the A1c-Derived Average Glucose (ADAG) study to develop a more precise prediction equation for estimating mean chronic glucose levels [30]. The equation resulting from the ADAG investigation is the one primarily used today. The study showed that 84% of the variance in mean glucose levels could be explained by the prediction equation based on A1c, a much stronger relationship than the one derived from the DCCT [30]. However, although better, the ADAG equation still resulted in substantial prediction error. For example, an A1c of 6.0% (42 mmol/mol) predicts a mean glucose level of 126 mg/dL (5.4 mmol/L), but the 95% confidence interval (CI) is 100–152 mg/dL (5.5–8.5 mmol/L). Similarly, an A1c of 8.0% (64 mmol/mol) results in an estimated mean glucose level of 183 mg/dL (10.2 mmol/L), with a 95% CI of 147–217 mg/dL (8.1–12.1 mmol/L). Note that the predicted value of 183 mg/dL (10.2 mmol/L) is probably not the true value. The true value likely falls somewhere between 147 and 217 mg/dL (8.1–12.1 mmol/L), and there is roughly a 5% chance that the true value falls outside of the 147–217 mg/dL (8.1–12.1 mmol/L) interval. Given a specific A1c value, how many consider the large range within which the true predicted glucose value might fall?

The A1c test can be performed any time of the day and without the need of fasting. These are significant benefits. Moreover, the A1c assay is designed to produce results representing chronic blood glucose levels over a 2–3 month period. This is also a desirable quality, although the error associated with the predictive utility of the A1c is very high. Because of its purported qualities, many medical practitioners use the A1c instead of the OGTT to diagnose diabetes [31]. However, given the poor agreement between the OGTT and the A1c, based on the present national sample and other investigations, use of the A1c over the OGTT may not be a good choice. Even though the A1c is faster, easier, and less expensive to administer, it results in false negative findings 60–70% of the time when the OGTT detects diabetes. How much is a missed diagnosis worth? Clearly, diabetes classifications resulting from the two assessments disagree far more than they agree.

So why do classifications resulting from the OGTT and the A1c disagree frequently? It is likely because there are many factors that influence A1c results other than glucose metabolism [32,33]. The World Health Organization (WHO) reports that A1c levels can be affected by genetic, hematologic, and illness-related factors, especially anemia [32]. According to Gallagher, erythropoiesis, the production of red blood cells, can be a significant factor affecting A1c [33]. Moreover, deficiencies in iron or vitamin B12 can lead to decreased erythropoiesis and increased A1c. On the other hand, administration of iron, vitamin B12, or erythropoietin; reticulocytosis, and chronic liver disease, can decrease A1c [33]. Moreover, large amounts of aspirin, excess alcohol consumption, chronic opiate use, hyperbilirubinemia, carbamylated hemoglobin, and increased lifespan of erythrocytes, can increase A1c levels, and hypertriglyceridemia can decrease A1c [33]. Additionally, chemical or genetic alterations of hemoglobin, liver failure, altered intra-erythrocyte pH, diseases of the spleen, rheumatoid arthritis, the glycation gap, and a variety of prescription drugs, can influence A1c levels [33,34,35,36,37,38]. Acute illness can also have an effect. In other words, although chronic blood glucose concentrations influence A1c levels, many other factors can increase or decrease A1c as well, which could lead to questionable A1c results and poor alignment with the OGTT. 

As seen in the present study of 7412 adults, diabetes classifications derived from the A1c assay frequently do not agree with the OGTT. Despite this concern, the A1c test appears to be a good predictor of disease and premature death. Research by Colagiuri et al., using a pooled analysis of 9 studies with over 28,000 individuals, reported that A1c was a significant predictor of diabetic retinopathy [39]. A recent study by Mancini et al. demonstrated that A1c was a significant predictor of survival within a sample of high-risk patients followed for 7 years with stable ischemic heart disease and diabetes [40]. Furthermore, a paper by Mo et al. indicated that A1c accounted for significant differences in macrovascular complications within a sample of Chinese individuals with Type 2 diabetes [41]. 

In a 15-year prospective cohort study, Selvin et al. showed that elevated A1c levels increased risk of all-cause mortality [42]. The investigation indicated that low levels of A1c (<5% or <31 mmol/mol) also increased risk of death from any cause. Overall, the study found that the baseline A1c measure was a better predictor of future disease and death than fasting glucose levels. 

In a case–cohort analysis, research by Nathan et al. showed that A1c was related significantly with retinopathy, nephropathy, and CVD [43]. Additionally, Forrest KY, Becker DJ 2000, found that A1c increased risk of developing lower-extremity arterial disease (LEAD) over a six-year follow-up period [44]. Lastly, A1c was related significantly with carotid intima-media wall thickness, after adjusting for several covariates, in a large, multi-ethnic, cross-sectional investigation by McNeely et al. Overall, it seems clear that elevated levels of A1c increase risk of numerous diseases. 

Although many investigations indicate that elevated levels of A1c increase risk of disease, not all studies support the link. Pruzin et al. found no association between A1c and global or regional Alzheimer disease pathology [45]. In a prospective cohort study by Orchard et al., after 10 years of follow-up, baseline A1c showed no relationship with CAD [46]. Moreover, in a cross-sectional investigation conducted in 31 centers in 16 European countries, research by Koivisto et al. identified no association between A1c and CVD [47].

### 4.2. Comparing the OGTT and FPG

Concordance between the OGTT and fasting plasma glucose (FPG) was better than the agreement between the OGTT and the A1c assay. This is probably because the OGTT and FPG are the same test administered under different conditions. The A1c assay is an entirely different assessment and does not measure glucose metabolism directly. Moreover, estimation of chronic glucose levels from A1c values are based on a linear regression equation with significant prediction error.

In the present study, when the OGTT classified an individual as diabetic with the entire sample included in the analysis (*n* = 7412), the FPG test resulted in the same classification 44.3% of the time—worse than a coin-flip. Delimited to women only, agreement was lower, 33.7%. The FPG test was more likely to classify a woman as prediabetic (52.8%) than diabetic (33.7%) when the OGTT labeled her diabetic. Confined to men, concordance was about 60%. With the focus on older adults only, the FPG test agreed with an OGTT classification of diabetes only 33.5% of the time.

Despite the lack of agreement between diabetes classifications based on the OGTT and fasting plasma glucose, many investigations show that the FPG test is a good predictor of disease and premature death. For example, in an investigation including 97 prospective studies, fasting glucose levels were shown to be predictive of increased risk of cancer death (43 studies), vascular mortality (50 investigations), and noncancer, nonvascular death (42 studies) [48]. In another meta-analysis, including 102 prospective investigations and approximately 280,000 individuals, Sarwar et al. reported that fasting blood glucose was non-linearly related to vascular risk and linearly associated with CHD [49]. Additionally, Park et al. studied almost 1.2 million Korean adults prospectively for 16 years and reported that as fasting glucose levels increased, ischemic heart disease, myocardial infarction, and thrombotic stroke increased in a J-shaped fashion [50].

### 4.3. Sensitivity and Specificity

Sensitivity is the ability of a test (e.g., A1c) to correctly identify individuals with a specific disease (diabetes). It is sometimes called the true positive rate. Specificity is the ability of a test to correctly identify those who do not have the disease, the true negative rate. Sensitivity and specificity are both important. In the present study, diagnosis of diabetes and prediabetes was based on results derived from the OGTT. 

Disease prevalence affects sensitivity and specificity rates. Because diabetes rates differ widely across age, relatively few young adults had the disease, whereas diabetes was more than 10 times higher in older adults. Risk of false positive results is higher when actual prevalence is low. 

Both the A1c assay and the FPG test had nearly perfect specificity or true negative rates. In other words, both were able to accurately identify adults who did not have diabetes or prediabetes. This was partly because the vast majority of adults in the sample did not have diabetes or prediabetes. One could predict that every adult in the present study had normal glucose metabolism and the results would have been a true negative rate of better than 75%. However, the A1c and the FPG tests both had low sensitivity or true positive rates, when administered alone, especially the A1c. With all 7412 adults included in the analysis, the A1c assay was able to detect those with diabetes only about one-third of the time and the FPG test had a true positive rate of only 44%. High specificity with low sensitivity is a dangerous combination in a clinical setting when the disease under consideration is diabetes.

### 4.4. Studies Comparing the FPG, A1c, and OGTT

When employed individually, the FPG and A1c assessments frequently fail to detect prediabetes and diabetes. Other studies have found similar outcomes. Using almost 500 adults from Finland, Saukkonen et al. compared the screening value of the FPG, A1c, and OGTT and concluded that concordance among the tests was not good [4]. Focusing mainly on the A1c test for diagnosing diabetes and prediabetes, Guo et al. determined that the assessment, when employed alone, had high false-negative results (65–75%) [5]. The authors indicated that the test “fails to identify a significant portion of patients” with diabetes or prediabetes, similar to the findings of the present study. When using the A1c and FPG together, Guo et al. found an unacceptable false-negative rate of 46%. Cowie et al. conducted a similar study and concluded that the A1c assessment substantially underestimates the prevalence of undiagnosed and total diabetes compared to the FPG or OGTT [6]. Moreover, a pooled analysis with over 300,000 subjects found similar results [7]. Finally, in a study with several hundred black African participants, Briker et al. showed that the A1c test was able to identify only 32% of diabetic cases. The authors concluded that the A1c assessment “underperforms as a diagnostic test” [8].

### 4.5. Predictive Power of the FPG and A1c Tests When Used Together

As individual assessments for detecting prediabetes and diabetes, the FPG and A1c tests are of questionable value, especially the latter. Under many conditions, a coin flip is a better predictor. According to the present study, the FPG and A1c assessments also perform poorly when combined, if the goal is to identify prediabetes. Even when both tests indicate prediabetes, agreement with the OGTT is less than 33%. However, when used together, and when both point to a diagnosis of diabetes, agreement with the OGTT is high (93%). Similarly, when both tests classify adults as normal, concordance is also 93%. 

Clearly, when the FPG or A1c assessments are used as single tests, there is a high probability that the resulting classification will be false. Administering both tests is valuable under two conditions: when both tests detect diabetes, or both conclude normal glycemia. Otherwise, their predictive utility is low and unsatisfactory compared to the OGTT.

### 4.6. Limitations and Strengths

The present study had several limitations. First, diagnosis of diabetes typically requires two abnormal test outcomes from the same blood sample [51] or from two different test samples [52]. Results of the present investigation were based on single tests for the OGTT, A1c, and FPG. Increased concordance may have occurred if a confirming test was administered after a diagnosis of diabetes or prediabetes. Additionally, fasting was self-reported. A fasting questionnaire was used to identify individuals who failed to fast legitimately, but a partial fast could have been hidden from NHANES. A partial fast could also be hidden from clinicians. An illegitimate fast would have affected the OGTT and FPG results. 

There were also multiple strengths associated with the present investigation. First, the study sample was large (*n* = 7412), multiracial, and selected randomly. In addition, participants were assigned individual sample weights by NHANES. The sample weights were included as part of each analysis, allowing the results to be generalized to all civilian, non-institutionalized adults in the United States. Second, the OGTT, A1c, and FPG assays were performed using high quality measurement methods. Third, women and men were studied separately and combined. Moreover, young adults, middle-age adults, and older adults were evaluated separately. A final strength was that the predictive value of combining the FPG and A1c results for detecting normal glycemia, prediabetes, and diabetes was compared to the OGTT. 

### 4.7. Future Research

Given the magnitude of the diabetes problem in developed countries, it is critical that issues associated with the detection of prediabetes and diabetes be resolved. Ease of administration and low cost are of little value if diagnostic tests are not accurate. Realigning the FPG and A1c test cut-points warrants evaluation beyond previous research. Investigations focusing on physiological and lifestyle factors that improve the predictive utility of the FPG and A1c tests are also warranted. Investigations designed to determine the cost-effectiveness of the OGTT, A1c, and FPG tests used in different combinations are also needed, since the OGTT is a better assessment, but more difficult and costly to administer. 

## 5. Conclusions

In conclusion, given current definitions of diabetes, prediabetes, and normal glucose metabolism, the present investigation shows that the OGTT, A1c, and FPG test results are out of alignment. With the OGTT as the standard, the A1c and FPG assessments have low sensitivity. Under some conditions, the A1c test has remarkably low sensitivity. When employed as lone tests, the odds of false negative outcomes are very high when using the FPG or A1c. These common tests frequently indicate that adults have normal glucose levels when they are prediabetic, or they classify individuals as prediabetic when they are diabetic. This is dangerous. Misdiagnosis can lead to catastrophic consequences. Clinicians should be extremely wary of administering the FPG or A1c tests as isolated assessments. Using both the FPG and A1c can improve diagnostic accuracy for detecting diabetes and normal glycemia, if there is complete agreement between the two tests. Individually or combined, the FPG and A1c assessments often misdiagnose prediabetes. Although more difficult to administer and more costly, use of the OGTT leads to far fewer diagnostic errors. 

## Figures and Tables

**Table 1:** Percentiles with standard errors for continuous variables in U.S. women (*n* = 3809) and men (*n* = 3603), and combined data (*n* = 7412).

Variable	Percentile (±SE)				
	5th	25th	50th	75th	95th
**2 h OGTT**					
Women (mg/dL)	68.0 ± 1.3	87.8 ± 0.6	105.1 ± 0.8	131.6 ± 1.2	203.0 ± 2.9
Women (mmol/L)	3.77 ± 0.07	4.87 ± 0.03	5.83 ± 0.04	7.30 ± 0.07	11.27 ± 0.16
Men (mg/dL)	58.6 ± 1.3	84.7 ± 0.7	103.1 ± 0.9	129.0 ± 1.1	198.0 ± 4.8
Men (mmol/L)	3.25 ± 0.07	4.70 ± 0.04	5.72 ± 0.05	7.16 ± 0.06	11.0 ± 0.27
Combined (mg/dL)	62.4 ± 1.0	86.1 ± 0.6	104.3 ± 0.6	130.4 ± 0.8	201.0 ± 2.7
Combined (mmol/L)	3.46 ± 0.06	4.78 ± 0.03	5.79 ± 0.03	7.24 ± 0.04	11.2 ± 0.15
**A1c**					
Women (%)	4.8 ± 0.02	5.1 ± 0.01	5.4 ± 0.01	5.6 ± 0.01	6.1 ± 0.02
Women (mmol/mol)	29 ± 0.12	32 ± 0.06	36 ± 0.07	38 ± 0.07	43 ± 0.14
Men (%)	4.7 ± 0.02	5.1 ± 0.01	5.4 ± 0.01	5.6 ± 0.01	6.1 ± 0.04
Men (mmol/mol)	28 ± 0.12	32 ± 0.06	36 ± 0.07	38 ± 0.07	43 ± 0.29
Combined (%)	4.8 ± 0.01	5.1 ± 0.01	5.4 ± 0.01	5.6 ± 0.01	6.1 ± 0.02
Combined (mmol/mol)	29 ± 0.06	32 ± 0.06	36 ± 0.07	38 ± 0.07	43 ± 0.14
**Fasting Plasma Glucose**					
Women (mg/dL)	81.7 ± 0.3	89.6 ± 0.2	95.4 ± 0.3	102.7 ± 0.4	118.8 ± 1.0
Women (mmol/L)	4.53 ± 0.02	4.97 ± 0.01	5.29 ± 0.02	5.70 ± 0.02	6.59 ± 0.06
Men (mg/dL)	85.6 ± 0.6	93.6 ± 0.3	99.6 ± 0.3	106.8 ± 0.4	124.0 ± 1.0
Men (mmol/L)	4.75 ± 0.03	5.19 ± 0.02	5.53 ± 0.02	5.93 ± 0.02	6.88 ± 0.06
Combined (mg/dL)	82.8 ± 0.3	91.4 ± 0.2	97.6 ± 0.2	104.9 ± 0.3	121.1 ± 0.8
Combined (mmol/L)	4.60 ± 0.02	5.07 ± 0.01	5.42 ± 0.01	5.82 ± 0.02	6.72 ± 0.04
**Age (years)**					
Women	21.5 ± 0.3	31.6 ± 0.5	46.1 ± 0.6	59.3 ± 0.4	77.3 ± 0.7
Men	21.3 ± 0.2	30.9 ± 0.4	43.2 ± 0.7	57.3 ± 0.6	74.2 ± 0.6
Combined	21.4 ± 0.8	31.2 ± 0.4	44.6 ± 0.6	58.4 ± 0.2	76.0 ± 0.5
**Body weight (kg)**					
Women	50.3 ± 0.4	61.7 ± 0.4	71.9 ± 0.5	86.3 ± 0.8	115.6 ± 1.7
Men	61.6 ± 0.5	74.6 ± 0.5	85.8 ± 0.7	99.5 ± 0.8	125.1 ± 1.6
Combined	53.4 ± 0.4	66.7 ± 0.3	79.1 ± 0.4	93.8 ± 0.5	121.7 ± 1.1

SE is standard error of the percentage.

**Table 2:** Agreement between test results for the Oral glucose tolerance test (OGTT) and the hemoglobin test (A1c) in U.S. women and men analyzed separately and combined, 2009–2016.

OGTT Classification	A1c Classification		
**All Subjects**	**A1c: Normal**	**A1c: Prediabetes**	**A1c: Diabetes**
OGTT: Normal	*n* = 4295	*n* = 1370	*n* = 13
*n* = 5678	row %: 80.7	row %: 19.2	row %: 0.1
OGTT: Prediabetes	*n* = 581	*n* = 599	*n* = 41
*n* = 1221	row %: 51.8	row %: 46.1	row %: 2.1
OGTT: Diabetes	*n* = 83	*n* = 244	*n* = 186
*n* = 513	row %: 18.6	row %: 47.3	row %: 34.1
Column: *n* = 7412	Column: *n* = 4959	Column: *n* = 2213	Column: *n* = 240
**Women Only**	**A1c: Normal**	**A1c: Prediabetes**	**A1c: Diabetes**
OGTT: Normal	*n* = 2231	*n* = 686	*n* = 5
*n* = 2922	row %: 80.4	row %: 19.5	row %: 0.1
OGTT: Prediabetes	*n* = 298	*n* = 306	*n* = 21
*n* = 625	row %: 51.1	row %: 46.4	row %: 2.5
OGTT: Diabetes	*n* = 47	*n* = 129	*n* = 86
*n* = 262	row %: 20.9	row %: 49.5	row %: 29.5
Column: *n* = 3809	Column: *n* = 2576	Column: *n* = 1121	Column: *n* = 112
**Men Only**	**A1c: Normal**	**A1c: Prediabetes**	**A1c: Diabetes**
OGTT: Normal	*n* = 2064	*n* = 684	*n* = 8
*n* = 2756	row %: 81.1	row %: 18.8	row %: 0.1
OGTT: Prediabetes	*n* = 283	*n* = 293	*n* = 20
*n* = 596	row %: 52.6	row %: 45.7	row %: 1.7
OGTT: Diabetes	*n* = 36	*n* = 115	*n* = 100
*n* = 251	row %: 15.6	row %: 44.6	row %: 39.8
Column: *n* = 3603	Column: *n* = 2383	Column: *n* = 1092	Column: *n* = 128

Note: SE: standard error of the weighted Kappa. OGTT: Oral glucose tolerance test. For the 2-h OGTT, “Normal” was defined as a plasma glucose level of <140 mg/dL (<7.8 mmol/L), “Prediabetes” was 140 mg/dL (7.8 mmol/L) to 199 mg/dL (11.0 mmol/L), and “Diabetes” was defined as a 2-h glucose level ≥ 200 mg/dL (11.1 mmol/L). For the A1c test, “Normal” was <5.7% (<39 mmol/mol), “Prediabetes” was 5.7–6.4% (39–47 mmol/mol), and a diagnosis of “Diabetes” was defined as an A1C ≥ 6.5% (≥48 mmol/mol). Because National Health and Nutrition Examination Survey (NHANES) sample weights were applied to each participant, the sample size of each category should be interpreted using row percentages, which have been adjusted based on individual sample weights, not “*n*”.

**Table 3:** Agreement between diabetes classifications based on the OGTT and the A1c in U.S. adults analyzed by age group, 2009–2016.

OGTT Classification	A1c Classification		
**Young Adults**	**A1c: Normal**	**A1c: Prediabetes**	**A1c: Diabetes**
OGTT: Normal	*n* = 2117	*n* = 282	*n* = 1
*n* = 2400	row %: 90.3	row %: 9.7	row %: 0.0
OGTT: Prediabetes	*n* = 150	*n* = 60	*n* = 3
*n* = 569	row %: 72.9	row %: 26.1	row %: 1.0
OGTT: Diabetes	*n* = 9	*n* = 16	*n* = 31
*n* = 292	row %: 19.5	row %: 34.1	row %: 46.4
Column: *n* = 2669	Column: *n* = 2276	Column: *n* = 358	Column: *n* = 35
**Middle-age Adults**	**A1c: Normal**	**A1c: Prediabetes**	**A1c: Diabetes**
OGTT: Normal	*n* = 1441	*n* = 566	*n* = 6
*n* = 2013	row %: 77.8	row %: 22.1	row %: 0.2
OGTT: Prediabetes	*n* = 204	*n* = 220	*n* = 15
*n* = 439	row %: 52.1	row %: 45.9	row %: 2.0
OGTT: Diabetes	*n* = 30	*n* = 57	*n* = 78
*n* = 165	row %: 22.5	row %: 35.4	row %: 42.1
Column: *n* = 2617	Column: *n* = 1675	Column: *n* = 843	Column: *n* = 99
**Older Adults**	**A1c: Normal**	**A1c: Prediabetes**	**A1c: Diabetes**
OGTT: Normal	*n* = 737	*n* = 522	*n* = 6
*n* = 1265	row %: 64.0	row %: 35.9	row %: 0.2
OGTT: Prediabetes	*n* = 227	*n* = 319	*n* = 23
*n* = 569	row %: 41.3	row %: 55.8	row %: 2.8
OGTT: Diabetes	*n* = 44	*n* = 171	*n* = 77
*n* = 292	row %: 15.5	row %: 59.2	row %: 25.2
Column: *n* = 2126	Column: *n* = 1008	Column: *n* = 1012	Column: *n* = 106

Note: SE: standard error of the weighted Kappa. OGTT: Oral glucose tolerance test. For the 2-h OGTT, “Normal” was defined as a plasma glucose level of <140 mg/dL (<7.8 mmol/L), “Prediabetes” was 140 mg/dL (7.8 mmol/L) to 199 mg/dL (11.0 mmol/L), and “Diabetes” was defined as a 2-h glucose level ≥ 200 mg/dL (11.1 mmol/L). For the A1c test, “Normal” was <5.7% (<39 mmol/mol), “Prediabetes” was 5.7–6.4% (39–47 mmol/mol), and a diagnosis of “Diabetes” was defined as an A1C ≥ 6.5% (≥48 mmol/mol). “Young adults” was defined as adults 20–39 years old. “Middle-age” included adults 40–59 years of age. “Older adults” was defined as individuals 60–80 years old. Because NHANES sample weights were applied to each participant, the sample size of each category should be interpreted using row percentages, which have been adjusted based on individual sample weights, not “*n*”. Results using “*n*” do not reflect the effect of the NHANES sample weights.

**Table 4:** Agreement between diabetes classifications for the OGTT and the fasting plasma glucose (FPG) test in U.S. women and men analyzed separately and combined, 2009–2016.

OGTT Classification	Fasting Plasma Glucose Classification		
**All Subjects**	**FPG: Normal**	**FPG: Prediabetes**	**FPG: Diabetes**
OGTT: Normal	*n* = 3595	*n* = 2043	*n* = 40
*n* = 5678	row %: 64.8	row %: 34.7	row %: 0.5
OGTT: Prediabetes	*n* = 381	*n* = 758	*n* = 82
*n* = 1221	row %: 30.9	row %: 63.4	row %: 5.8
OGTT: Diabetes	*n* = 37	*n* = 248	*n* = 228
*n* = 513	row %: 8.9	row %: 46.8	row %: 44.3
Column: *n* = 7412	Column: *n* = 4013	Column: *n* = 3049	Column: *n* = 350
**Women Only**	**FPG: Normal**	**FPG: Prediabetes**	**FPG: Diabetes**
OGTT: Normal	*n* = 2130	*n* = 781	*n* = 11
*n* = 2922	row: 73.4%	row: 26.3%	row: 0.3%
OGTT: Prediabetes	*n* = 232	*n* = 360	*n* = 33
*n* = 625	row: 36.8%	row: 59.2%	row: 4.0%
OGTT: Diabetes	*n* = 30	*n* = 143	*n* = 89
*n* = 262	row: 13.5%	row: 52.8%	row: 33.7%
Column: *n* = 3809	Column: *n* = 2392	Column: *n* = 1284	Column: *n* = 133
**Men Only**	**FPG: Normal**	**FPG: Prediabetes**	**FPG: Diabetes**
OGTT: Normal	*n* = 1465	*n* = 1262	*n* = 29
*n* = 2756	row: 55.3%	row: 43.8%	row: 0.8%
OGTT: Prediabetes	*n* = 149	*n* = 398	*n* = 49
*n* = 596	row: 24.3%	row: 68.0%	row: 7.7%
OGTT: Diabetes	*n* = 7	*n* = 105	*n* = 139
*n* = 251	row: 3.0%	row: 39.0%	row: 57.9%
Column: *n* = 3603	Column: *n* = 1621	Column: *n* = 1765	Column: *n* = 217

Note: SE: standard error of the weighted Kappa. For the 2-h OGTT, “Normal” was defined as a plasma glucose level of <140 mg/dL (<7.8 mmol/L), “Prediabetes” was 140–199 mg/dL (7.8–11.0 mmol/L), and “Diabetes” was defined as a glucose level ≥ 200 mg/dL (11.1 mmol/L). For the FPG (fasting plasma glucose) assessment, “Normal” was <100 mg/dL (<7.0 mmol/L), “Prediabetes” was 100–125 mg/dL (5.6–6.9 mmol/L), and a diagnosis of “Diabetes” was defined as a glucose level ≥ 126 mg/dL (≥7.0 mmol/L). Because NHANES sample weights were applied to each participant, the sample size of each category should be interpreted using row percentages, which have been adjusted based on individual sample weights, not “*n*”. Results using “*n*” do not reflect the effect of the NHANES sample weights.

**Table 5:** Agreement between test results for the OGTT and FPG in U.S. adults analyzed by age group, 2009–2016.

OGTT Classification	Fasting Plasma Glucose Classification		
**Young Adults**	**FPG: Normal**	**FPG: Prediabetes**	**FPG: Diabetes**
OGTT: Normal	*n* = 1760	*n* = 632	*n* = 8
*n* = 2400	row %: 73.8	row %: 26.0	row %: 0.2
OGTT: Prediabetes	*n* = 93	*n* = 112	*n* = 8
*n* = 213	row %: 43.8	row %: 52.4	row %: 3.8
OGTT: Diabetes	*n* = 6	*n* = 16	*n* = 34
*n* = 56	row %: 14.6	row %: 31.4	row %: 54.0
Column: *n* = 2669	Column: *n* = 1859	Column: *n* = 760	Column: *n* = 50
**Middle-age Adults**	**FPG: Normal**	**FPG: Prediabetes**	**FPG: Diabetes**
OGTT: Normal	*n* = 1186	*n* = 808	*n* = 19
*n* = 2013	row %: 60.4	row %: 38.8	row %: 0.8
OGTT: Prediabetes	*n* = 146	*n* = 260	*n* = 33
*n* = 439	row %: 31.3	row %: 62.6	row %: 6.1
OGTT: Diabetes	*n* = 9	*n* = 64	*n* = 92
*n* = 165	row %: 8.0	row %: 35.9	row %: 56.1
Column: *n* = 2617	Column: *n* = 1341	Column: *n* = 1132	Column: *n* = 144
**Older Adults**	**FPG: Normal**	**FPG: Prediabetes**	**FPG: Diabetes**
OGTT: Normal	*n* = 649	*n* = 603	*n* = 13
*n* = 1265	row %: 51.9	row %: 47.2	row %: 0.9
OGTT: Prediabetes	*n* = 142	*n* = 386	*n* = 41
*n* = 569	row %: 24.2	row %: 69.5	row %: 6.3
OGTT: Diabetes	*n* = 22	*n* = 168	*n* = 102
*n* = 292	row %: 8.2	row %: 58.4	row %: 33.5
Column: *n* = 2126	Column: *n* = 813	Column: *n* = 1157	Column: *n* = 156

Note: SE: standard error of the weighted Kappa. For the 2-h OGTT, “Normal” was defined as a plasma glucose level of <140 mg/dL (<7.8 mmol/L), “Prediabetes” was 140–199 mg/dL (7.8–11.0 mmol/L), and “Diabetes” was defined as a glucose level ≥ 200 mg/dL (11.1 mmol/L). For the FPG (fasting plasma glucose) assessment, “Normal” was <100 mg/dL (<7.0 mmol/L), “Prediabetes” was 100–125 mg/dL (5.6–6.9 mmol/L), and a diagnosis of “Diabetes” was defined as a glucose level ≥ 126 mg/dL (≥7.0 mmol/L). “Young adults” was defined as adults 20–39 years old. “Middle-age” included adults 40–59 years of age. “Older adults” was defined as individuals 60–80 years old. Because NHANES sample weights were applied to each participant, the sample size of each category should be interpreted using row percentages, not “*n*.”.

## Data Availability

All NHANES data sets are available online for free to everyone [9].
